# Endophytic microbiome of *Boehmeria nivea* and their antagonism against latent fungal pathogens in plants

**DOI:** 10.1186/s12866-022-02737-1

**Published:** 2022-12-24

**Authors:** Asri Peni Wulandari, Erin Triani, Kartika Sari, Mila Prasetyani, Mohamad Nurzaman, Rully Dyah Purwati, Riksfardini A. Ermawar, Anne Nuraini

**Affiliations:** 1 Department of Biology, Faculty of Mathematics and Natural Sciences, Padjadjaran University, Sumedang, Indonesia; 2 Center for Bioprospection of Natural Fibers and Biological Resources, Faculty of Mathematics and Natural Sciences, Padjadjaran University, Sumedang, Indonesia; 3 Research Center of Sweetener Plants and Fibers, Ministry of Agriculture, Jakarta, Indonesia; 4 Research and Development Center of Biomaterials, National Research and Innovation Agency, Cibinong, Indonesia; 5 Department of Agrotechnology, Faculty of Agriculture, Padjadjaran University, Sumedang, Indonesia

**Keywords:** Antifungal, Antagonistic interaction, Endophyte, Microbiome, Ramie

## Abstract

**Background:**

Pathogenic microbes still become obstacles that can reduce the quality of plant growth, including ramie (*Boehmeria nivea*) plants. The study identified the microbiome and antagonistic interaction of the endophytic community from the *B. nivea* is necessary to improve the production of the ramie plant, especially ramie stem organs for fiber materials.

**Results:**

Twenty isolates of endophytic microorganisms were obtained from the roots, stems, leaves, and flowers. They were identified using the Internal Transcribed Spacer (ITS) region of ribosomal (rDNA), and its morphotypes obtained 20 isolates, with a composition of 9 species of bacteria and 11 species of fungi. Besides that, the disease observations on ramie stems showed that four species of pathogenic fungi were identified as *Fusarium solani* isolate 3,248,941, *Fusarium solani* isolates colpat-359, *Fusarium oxysporum* isolate N-61–2, *Clonostachys rosea* strain B3042. The endophytic microorganism of ramie ability was tested to determine their potential to inhibit the growth of the pathogenic fungi based on the in-vivo antagonist test. The isolated bacteria were only able to inhibit the growth of *F. solani*, with the highest percentage of 54–55%. Three species of endophytic fungi, including *Cladosporium tennissimum, Fusarium falciforme, and Penicillium citrinum*, showed the best inhibition against the fungal pathogen *Fusarium solani* with the highest inhibitory presentation of 91–95%. Inhibitory interaction between the endophytic microbes and the ramie pathogens indicated the type of antibiosis, competition, and parasitism.

**Conclusion:**

The results of this study succeeded in showing the potential antifungal by endophytic fungi from ramie against the pathogens of the plant itself. *P. citrinum* isolate MEBP0017 showed the highest inhibition against all the pathogens of the ramie.

## Background

Plants are constantly involved in interactions with various microbes that can promote the maintenance of biodiversity and the ecosystem [1]. One of the interactions is plants with endophytic microbes, living organisms in all healthy plant tissues without causing disease symptoms or morphological changes in plants [2]. Plant hosts protect the endophytic life cycle from environmental stresses and microbial competition [3].

Most endophytic fungi were found to be beneficial to their host plants and produce important bioactive compounds that have been used in many applications [4]. Endophytic bioactive compounds play an important role in ecology, the environment, and the medical field [5]. Endophytic microorganisms, closely related to host plants, can help plant growth and are useful for agricultural purposes. Besides increasing plant growth and resistance to biotic and abiotic stresses, such as pathogens, drought, and salinity, endophytes are also essential for drug discovery [6–9].

Endophytic microorganisms are distributed in various plant tissues such as the epidermis, mesophyll, palisade, parenchyma, vascular tissue, xylem and phloem, roots, stems, leaves, flowers, fruits, seeds, and pollen [10–15]. Posangi and Bara (2014) revealed that there are 300 thousand species of phanerogamae as hosts of endophytic microorganisms that live in plants contributing to producing some substances to be used as self-defense for host plants to survive. It makes endophytic organisms constantly evolve to produce new compounds to protect host plants [16].

It has been found that there is a variable relationship between endophytes and their host plants, such as mutualism, symbiosis, antagonism, and pathogenics [17]. Although some endophytes can be called pathogens, most are inactive within the host tissues. Some saprobes can also be facultative parasites. In addition, endophytic microorganisms tend to become pathogenic when the host plant is under stress conditions [18]. Especially in ramie plants, there has not been a complete study on the presence of endophytic microorganisms and their pathogens.

Ramie (*Boehmeria nivea*) is a source of natural fiber with unique fiber characteristics to be developed as a raw material for textiles, pulping, composites, and many more. This plant grows well in tropical and sub-tropical regions such as Southeast or South Asia and China. In the ramie cultivation system, farmers still face problems handling plant pests and diseases. The presence of plant diseases in the form of pathogenic fungi, especially in stem organs, can interfere with growth and reduce fiber productivity. Several pathogenic microorganisms such as *Rhizoctonia solani*, *Phytophthora megasperma*, *Aphanomyces euteiches*, *Macrophomina phaseolina, Clonostachys rosea*, and *Pythium sp* can cause significant losses in the land product [19].

Some plant pathogenic fungi can act as asymptomatic endophytes and adapt to their hosts before showing disease symptoms [20]. Some examples of latent pathogenic fungi that have been reported such *Colletotrichum gloeosporioides* (citrus anthracnose disease) [21], *Phomopsis citri*, *Lasiodiplodia theobromae, Botrypsphaeria* sp. (citrus stem tip rot disease) [22], *Sclerotinia pseudotuberosa* (black fruit rot) [23], and *Leptosphaerulina crassiasca* (leave pepper spots on peanuts) [24]. A study of fungal pathogens in ramie plants reported by Yu et al.(2016) stated that ramie root rot disease caused by *Phytopythium vexans* reduced crop productivity by up to 40% in South China [25]. Moreover, anthracnose disease is caused by *Colletotrichum gloeosporioides* and a leaf spot disease caused by *Cercospora boehmerae* [26].

Fungicide treatment cannot eliminate all endophytes. Due to some endophytes being latent pathogens, selecting healthy plants (asymptomatic) in the field for infection densities of latent pathogens and spraying with suitable fungicides can be important procedures in integrated pest management. Endophytic life cycle is protected from stressed environment and microbial competition by plant hosts [3]. Utilization of the presence of plant endophytes can interact with the pathogen. Accordingly, it gives them opportunities to develop biocontrols to overcome diseases caused by pathogenic microorganisms [27]. Ramie plants have endophytic microorganisms that have not been well explored, which can be used as a biocontrol against the plant pathogens. This study aims to explore the diversity of endophytic microorganisms present in various parts of ramie plants and also their antagonistic activity against ramie plant pathogenic microorganisms as an alternative biocontrol. The production of ramie fiber is highly dependent on the quality of the stems of the ramie plant. This study will specifically examine pathogenic fungi whose latent presence has not been identified, especially in the ramie stem organs.

## Results

### Microbiome endophytes of ramie

**Fig. 1:**
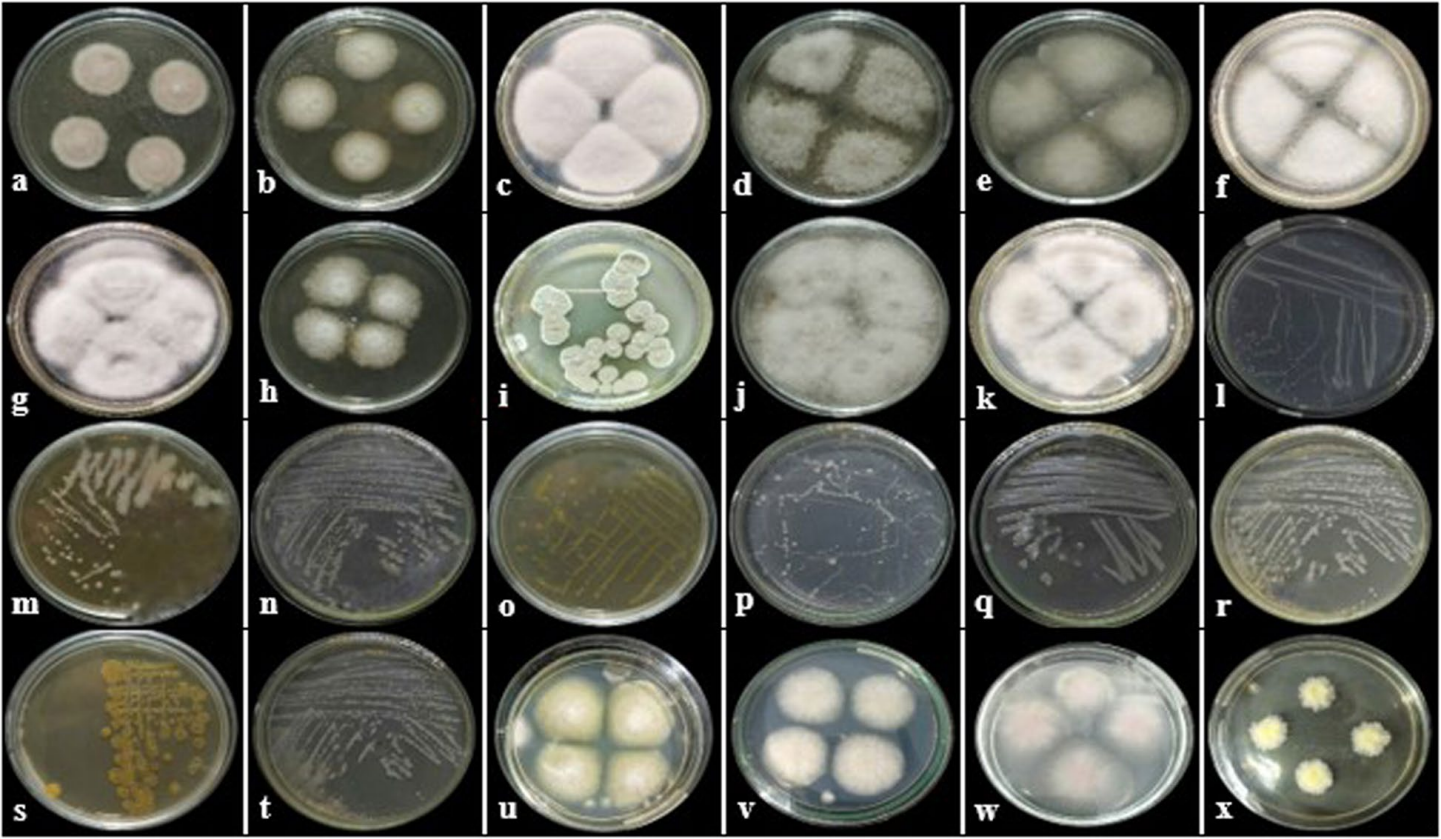
Morphology of endophytic fungi (**a**-**k**), endophytic bacteria (**l**–**t**), and pathogenic fungi (**u**-**x**) of ramie. Isolate codes: (**a**) EF-SS01, (**b**) EF-YLS02, (**c**) EF-FS01), (**d**) EF-FS03, (**e**) EF-YLS01, (**f**) EF-YLS04, (**g**) EF-YLS03, (**h**) EF-RS01, (**i**)EF-RS02, (**j**) EF-SS02, (**k**) EF-SS02; (**l**) EB-FS02, (**m**) EB-SS01, (**n**) EB-SS02, (**o**) EB-FS01, (**p**) EB-SS03, (**q**) EB-RS01, (**r**) EB-FS03, (**s**)EB-FS04, (**t**) EB-RS02; (**u**) PF-SS01, (**v**) PF-SS02, (**w**) PF-SS03, and (**x**) PF-SS04. EF = endophytic fungi; EB = endophytic bacteria; and PF = pathogenic fungi. Stem shoot (SS), rhizome shoots (RS), young leaf shoots (YLS), and flowers (FS)

In the endophytic microbiome from different parts of ramie, we found a total of 19 isolates: 11 endophytic fungi and 8 bacteria were isolated from rhizome shoots (RS), stem shoots (SS), young leaf shoots (YLS), and flowers (FS) of the plant. They were grouped according to their morphological characteristics of colony shape, mycelium color, and inverted media color (Fig. 1) to allow for the systematic selection of those isolates (Tables 1, 2 and 3). The endophytic fungi (EF) (Fig. 1: a-k) consisted: 4 isolates EF-YLS, 3 isolates EF-FS, 2 isolates EF-SS, and 2 isolates EF-RS. Most of the EF from ramie belong to the phylum Ascomycota while one species including Basidiomycota has been isolated as *Peniophora* sp isolate SAG15F1. Although, it is rarely found in endophytic fungal isolates [28, 29]. The endophytic bacteria (EB) (Fig. 1: l-t) consist of: 4 isolates EB-FS, 3 isolates EB-SS, and 1 isolate EB-RS, and no endophytic bacteria were found from leaf tissue.

**Table 1:** Morphology characteristic and molecular analysis of endophytic fungi (EF) Ramie

No	Organ of ramie plants	Isolate code	Species	Colony color		Radial line	Concen-tric circle	Colony shape	Edge of colony	Texture	Molecular Analysis			
				**Front side**	**Backside**						**Score**	**QC (%)**	**EV**	**MI (%)**
1	Rhizome shoots	EF-RS01	*Purpureocillium lilacinum*	White	Yellow	Pink	White	Circular	Entire	Cottony	1050	100	0,0	100%
2		EF-RS02	*Purpureocillium lilacinum*	Green	Yellow	-	-	Circular	Entire	Cottony	1050	100	0, 0	100
3	Stem shoots	EF-SS01	*Cladosporium tenuissimum* strain N1	Blackish-green	Yellow	-	-	Circular	Entire	Powdery	994	100	0, 0	100
4		EF-SS02	*Fusarium falciforme* strain DTO 421-G2	White	Yellow	-	-	Circular	Entire	Cottony	1037	100	0, 0	100
5	Flowers	EF-FS01	*Colletotrichum aenigma* isolate ZH2	White	White	-	-	Circular	Entire	Cottony	1046	100	0,0	100%
6		EF-FS02	*Fusarium falciforme* strain DTO 422-H8	White	Yellow	-	-	Circular	Entire	Cottony	1024	100	0,0	100%
7		EF-FS03	Fusarium falciforme strain DTO 422-H8	Blackish green	Yellow	-	-	Circular	Entire	Powdery	1024	100	0,0	100%
8	Young leaf shoots	EF-YLS01	*Colletotrichum gloeosporioides* isolate PL12	White	Yellow	-	-	Circular	Entire	Cottony	1053	99%	0,0	100%
9		EF-YLS02	*Penicillium citrinum* isolate MEBP0017	White	Yellow	-	-	Circular	Entire	Cottony	937	100	0,0	100%
10		EF-YLS03	*Peniophora* sp isolate SAG15F1	White	White	-	Green	Circular	Entire	Cottony	1064	100	0,0	100%
11		EF-YLS04	*Colletotrichum siamense* strain bl20	White	White	Black	-	Circular	Entire	Cottony	1046	100	0,0	99,82

**Table 2:** Morphology Characteristic and Molecular Analysis of Endophytic Bacteria (EB) Ramie

No	Organ of Ramie plants	Isolate Code	Species	Colony color		Gram test	Colony shape	Edge of colony	Texture	Molecular Analysis			
				**Front side**	**Backside**					**Score**	**QC (%)**	**EV**	**MI (%)**
1	Rhizome shoots	EB-RS01	*Stenotrophomonas* sp CanS-106	White	White	Negative	Circular	-	Slimy	2556	100	0,0	98,93
2		EB-RS02	*Cupriavidus pauculus* partial	Orange	Orange	Negative	Irregular	-	Slimy	2532	100	0,0	99,71
3	Stem shoots	EB-SS01	*Curtobacterium luteum* OsEp_Plm_15P7	Orange	Orange	Positive	Irregular	-	Slimy	2523	100	0,0	100
4		EB-SS02	*Curtobacterium citreum* strain OsEp_A&N_30A1	Orange	Orange	Positive	Irregular	-	Slimy	2528	100	0,0	100
5		EB-SS03	*Bacillus altitudinis*	White	White	Positive	Circular	-	Slimy	2564	100	0,0	100
6	Flowers	EB-FS01	*Bacillus haynesii* strain 1605	Yellow	Yellow	Positive	Irregular	-	Slimy	2524	100	0,0	99,78
7		EB-FS02	*Curtobacterium luteum* OsEp_Plm_15P7	Yellow	Yellow	Positive	Irregular	-	Slimy	2501	100	0,0	100
8		EB-FS03	*Pseudomonas psychrotolerans* strainOsEp_A&N_30A13	Yellow	Yellow	Negative	Circular	-	Slimy	2547	100	0,0	99,93
9		EBFS-4	*Stenotrophomonas* sp strain Atecer 6D*p*	White	White	Negative	Circular	-	Slimy	2490	100	0,0	99,93

**Table 3:** Morphology characteristic and molecular analysis of pathogenic fungi (PF) Ramie

No	Organ of ramie plants	Isolate code	Species	Colony color		Radial line	Concentric circle	Colony shape	Edge of colony	Texture	Molecular Analysis			
				**Front side**	**Backside**						**Score**	**QC (%)**	**EV**	**MI (%)**
1	Stem shoots	PF-SS01	*Fusarium solani* isolate 3,248,941	White	Yellow	White	White	Circular	Entire	Cottony	1050	100	0,0	100
2		PF-SS02	*Fusarium solani* isolate Colpat-359	White	Milky white	White	White	Circular	Entire	Cottony	1038	100	0,0	100
3		PF-SS03	*Fusarium oxysporum* isolate N-61–2	White	Milky white	White	purple	Circular	Entire	Cottony	989	100	0,0	99,8
4		PF-SS04	*Clonostachys rosea* strain B3042	White	Yellow	White	Yellow	Circular	Filiform	Cottony	1038	100	0,0	99,8

We also succeeded in isolating 4 isolates of pathogenic fungi (PF) (Fig. 1: u – x) consisted: *Fusarium* spp. (PF-SS01, PF-SS02, and PF-SS03) and one of species *Clonostachys rosea* (PF-SS04). *Fusarium* in the organ or tissue of ramie can be endophytic as well as pathogenic. As endophytic fungi, two EF as *F. falciforme* strain DTO 421-G2 and *F. falciforme* strain DTO 422-H8 could be isolated from the stem and flower tissues; on the other hand, FP consisted two species *F. solani*, and one PF as *F. oxysporum* can be identified different character of the colony from *F. solani*. It gave same morphological characteristics as the isolate *F. solani* Colpat-359 except for the difference in the purple concentric circles in the colonies.

### The phylogenetic of endophytic microorganisms and pathogen fungi

**Fig. 2:**
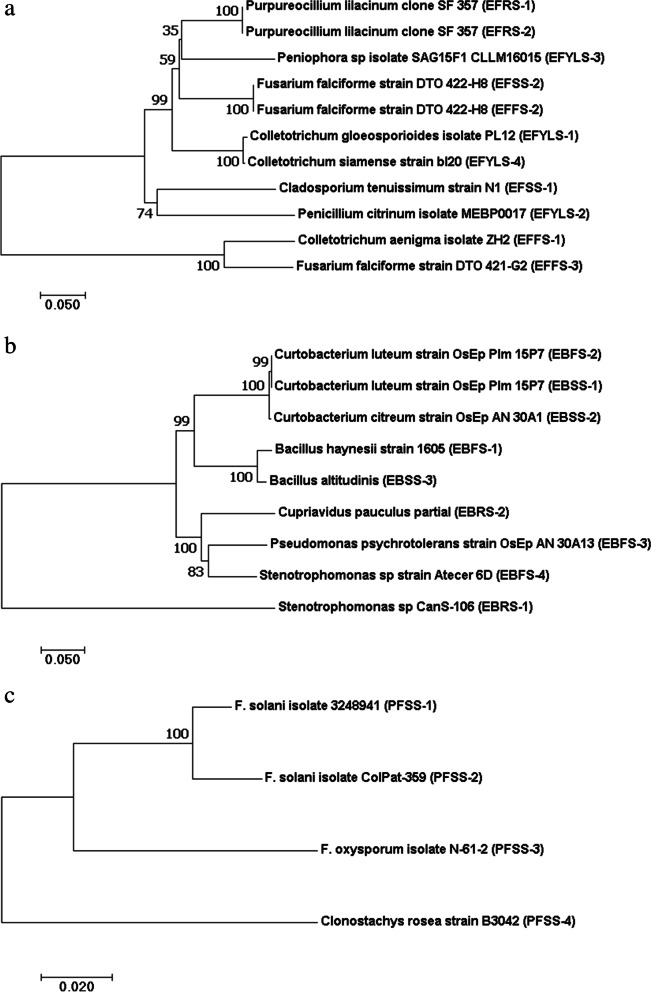
The phylogenetic tree of endophytic and pathogenic microbiome of *Boehmeria nivea* based on ITS gene with 1000 bootstrap replicates (**a**) fungi endophytic, (**b**) bacteria endophytic, and (**c**) fungi pathogenic

The identity of the ITS and 18S rDNA gene sequences obtained here and those available in GenBank ranged from 98 to 100%. Our results revealed that 11 endophytic fungi (EF) (Table 2), 9 endophytic bacteria (EB) (Table 3), and 4 fungi pathogens of the stem of ramie (PF) (Table 4) where a high diversity of plant-associated microbiome is expected. The topology of the phylogenetic tree of endophytic microbiome shows the presence of three main groups indicating their distribution in organelles (Fig. 2). Cladogram of EF with a neighbor-joining tree showed that the tree forms three clades to the phylum of Ascomycota, which includes three classes, including Sordariomycetes, Eurotiomycetes, and Dothideomycetes. Therefore, members of the phylum of Eumycophyta comprise only one class Deteromycetes, from the total identified specimens. Clade I consisted of 3 sub-group that have high similarity (100%), including *Colletotrichum gloeosporioides* isolate PL12 (EF-YLS01), dan *Colletotrichum siamense* strain bl20 (EF-YLS04).

**Table 4:** Antagonistic activity of endophytic fungi (EF) and endophytic bacteria (EB) against pathogenic fungi of ramie

**No**	**Species**		***F. solani* isolate 3,248,941**		***F. solani* isolate Colpat-359**		***F. oxysporum* isolate N-61–2**		***Clonostachys rosea* strain B3042**	
			**PGI (%)**	**Inter-action**	**PGI (%)**	**Inter-action**	**PGI (%)**	**Inter-action**	**PGI (%)**	**Inter-action**
1	*Purpureocillium lilacinum*	EF	82.44 (4)	P	83.05 (4)	P	79.38 (4)	P	64.70 (3)	P
2	*Cladosporium tenussimum* strain N1	EF	75.48 (4)	P	83.89 (4)	P	78.75 (4)	P	67.73 (3)	C
3	*Fusarium falciforme* strain DTO 422-H8	EF	82.63 (4)	C	82. 06 (4)	C	81.88 (4)	C	43.79 (2)	C
4	*Colletotrichum aenigma i*solate ZH2	EF	74.44 (3)	C	80.42 (4)	C	79.38 (4)	C	59.47 (3)	C
5	*Fusarium falciforme* strain DTO 421-G2	EF	78.49 (3)	C	78.27 (4)	C	86.88 (4)	C	62.27 (3)	C
6	*Colletotrichum gloeosporioides* isolate PL12	EF	81.23 (4)	P	92.07 (4)	C	83.13 (4)	C	71.29 (3)	C
7	*Penicillium citrinum* isolate MEBP0017	EF	81.73 (4)	A	86.01 (4)	A	86.25 (4)	A	82.95 (4)	A
8	*Peniophora sp isolate SAG15F*	EF	72.57 (3)	P	73.92 (3)	P	73.13 (3)	C	35.53 (2)	C
9	*Colletotrichum siamense strain bl20*	EF	68.45 (3)	P	75.00 (3)	P	69.38 (3)	P	46.74 (2)	P
10	*Stenotrophomonas* sp CanS-106	EB	54.23 (3)	P	51.62 (3)	A	49.07 (2)	P	13.65 (1)	A
11	*Stenotrophomonas sp* strain Atecer 6D	EB	54.28 (3)	P	51.77 (3)	A	49.09 (2)	A	13.29 (1)	C
12	*Curtobacterium luteum* OsEp_Plm_15P7	EB	54.16 (3)	C	51.60 (3)	P	49.22 (2)	C	13.61 (1)	P
13	*Bacillus haynesii* strain 1605	EB	55.07 (3)	C	52.24 (3)	C	49.85 (2)	C	14.83 (1)	C
14	*Pseudomonas psychrotolerans* strainOsEp_A&N_30A13	EB	54.49 (3)	C	51.90 (3)	P	49.18 (2)	P	12.60 (1)	A

Type of interaction: *A* antibiosis, *P* parasitic, *C* competition

Sub-group *Colletotrichum aenigma* isolate ZH2 (EF-FS01), and *Fusarium falciforme* strain DTO 421-G2 (EF-FS03) have high similarities. The sub-group with less similarity (74%) was *Cladosporium tenuissimum* strain N1 (EFSS-1), *Penicillium citrinum* isolate MEBP0017 (EF-YLS02); Clade II consisting of Peniophora sp. isolate SAG15F1 (EF-YLS03), and two species of *Purpureocillium lilacinum* isolated from rhizome tissue identified as *P. lilacinum* clone SF_357 (EF-RS01), and *P. lilacinum* strain ZMGRS3 (EF-RS02); and Clade III of species *Fusarium falciforme* strain DTO 422-H2 was isolated from different stem and flower tissues.

Bacterial microbiome that can be isolated from rhizome shoots, stems shoots, young leaf shoots, and flowers consisted of three phyla, including Proteobacteria (*Stenotrophomonas sp, Cupriavidus pauculus, Pseudomonas psychrotolerans*), Actinobacteria (*Curtobacterium luteum, C. citreum*), and Firmicutes (*Bacillus altitudinis, B. haynesii*). Based on the bootstrap phylogenetic tree of endophytic bacteria (Fig. 2b), it has relatively high sequence similarities 99% with accession of *Curtobacterium luteum* strain OsEp Plm 15P7 (EB-FS02) and *C. luteum* strain OsEp Plm 15P7 (EB-SS01) formed a sister clade; however, the two sequences formed a dichotomy against *C. citreum* strain OsEp AN 30A1 (EB-SS02). *Bacillus haynesii* strain 1605 (EB-FS01) and *B. altitudinis* (EB-SS03) formed a sister clade with the bootstrap value of both sequences of 100%. *Stenotrophomonas sp* CanS-106 (EB-RS01) is closely related to *C. luteum* strain OsEp Plm 15P7 (EB-FS02), *C. luteum* strain OsEp Plm 15P7 (EB-SS01), *C. citreum* strain OsEp AN 30A1 (EB-SS02), *Bacillus haynesii* strain 1605 (EB-FS01), and *Bacillus altitudinis* (EB-SS03) all showed the same relationships with strong bootstrap supports (99—100%). *Pseudomonas psychrotolerans* OsEp strain AN 30A13 (EB-FS03) and *Stenotrophomonas s*p strain Atecer 6D (EB-FS04) formed a sister clade with a bootstrap value of 83%. *Cupriviadus pauculus* partial (RB-RS02) as an out group which morphologically resembles one of the *Curtobacterium* strains even though the cell wall structure is gram negative, however this isolate was only found in the ramie root system.

Further phylogenetic analysis based on the ITS gene of fungi pathogens (Fig. 2c) can provide information about the molecular phylogenetic of *Fusarium* at the species level and strain for *F. solani* isolate 3,248,941 (PFSS-1), *F. solani* isolate Colpat-359 (PFSS-2), and *F. oxysporum* isolate N-61–3 (PFSS-3) with strong relationship, however construction places *Clonostachys rosea* as an outgroup.

### Antagonistic activity and Interaction Mechanism in-vitro

The inhibitory activity screening test turned out to be only 9 out of 11 fungal isolates (Tabel 4). The evaluation of the inhibitory potential was the PGI value [30, 31]. In general, the activity of the antagonist fungi showed inhibition for *Fusarium* spp. in the range PGI of 68.45—92.07 or in the category of values of 3—4 (strong to very strong). However, the activity slightly decreased on *C. gloeosporioides* with a value category only in the range of 2 -3, except for *P. citrinum* which showed category a very active for all the fungi pathogen. *Fusarium* spp. from the ramie seems to be more sensitive to the presence of endophytic microorganisms when compared to *Clonotachys rosea.*

The endophytic bacteria (EB) showed inhibitory activity screening test turned out to be only 5 out of 8 bacteria that antagonistic effects against fungal pathogens with a value category of 1–3 (weak to strong). The inhibitory effect of endophytic fungi shows greater potential than endophytic bacteria on ramie pathogens. Antagonistic activity of endophytic fungi showed a weaker against the pathogen *Clonostachys rosea* with a PGI range of 2–3 except for *Penicillium citrinum* isolate MEBP0017 with a PGI value of 4. *Penicillium citrinum* has a very active spectrum against all pathogens and can be recommended as a candidate for antifungal pathogens.

**Fig. 3:**
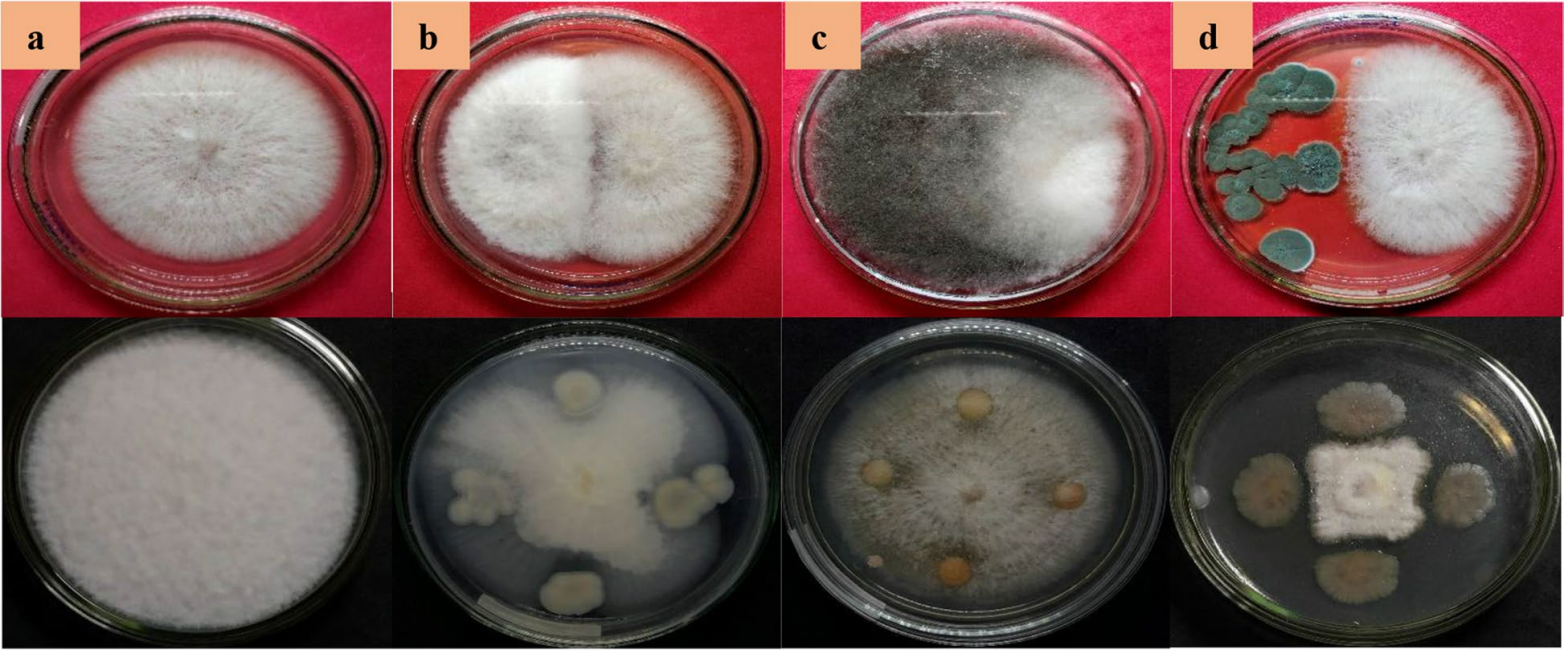
Interaction mechanism of endophytic fungi (above) and bacteria (below): (**a**) control; (**b**) parasitism; (**c**) competition; and (**d**) antibiosis

The type of mechanism that occurs during inhibition by endophytic microorganisms against pathogens can be observed based on the categories described by Skidmore and Dickinson [32]. Some endophytic fungi or bacteria show the type of competition if the fungal colony shows an antagonistic effect by covering the pathogenic colony and the growth of the antagonist fungus more quickly fills the surface; while an antibiosis can be demonstrated if an empty zone is formed between the pathogenic fungus and the antagonist fungus. This antagonistic effect generally indicates that there is a change in the shape of the hyphae of the pathogen or the formation of pigment on the lower surface of the fungal colony; whereas parasitism, when the antagonist fungus grows on the hyphae of the pathogen, in the contact area of the hyphae of the antagonist fungus is found wrapped around the hyphae of the pathogen and undergoes lysis (Fig. 3).

**Fig. 4:**
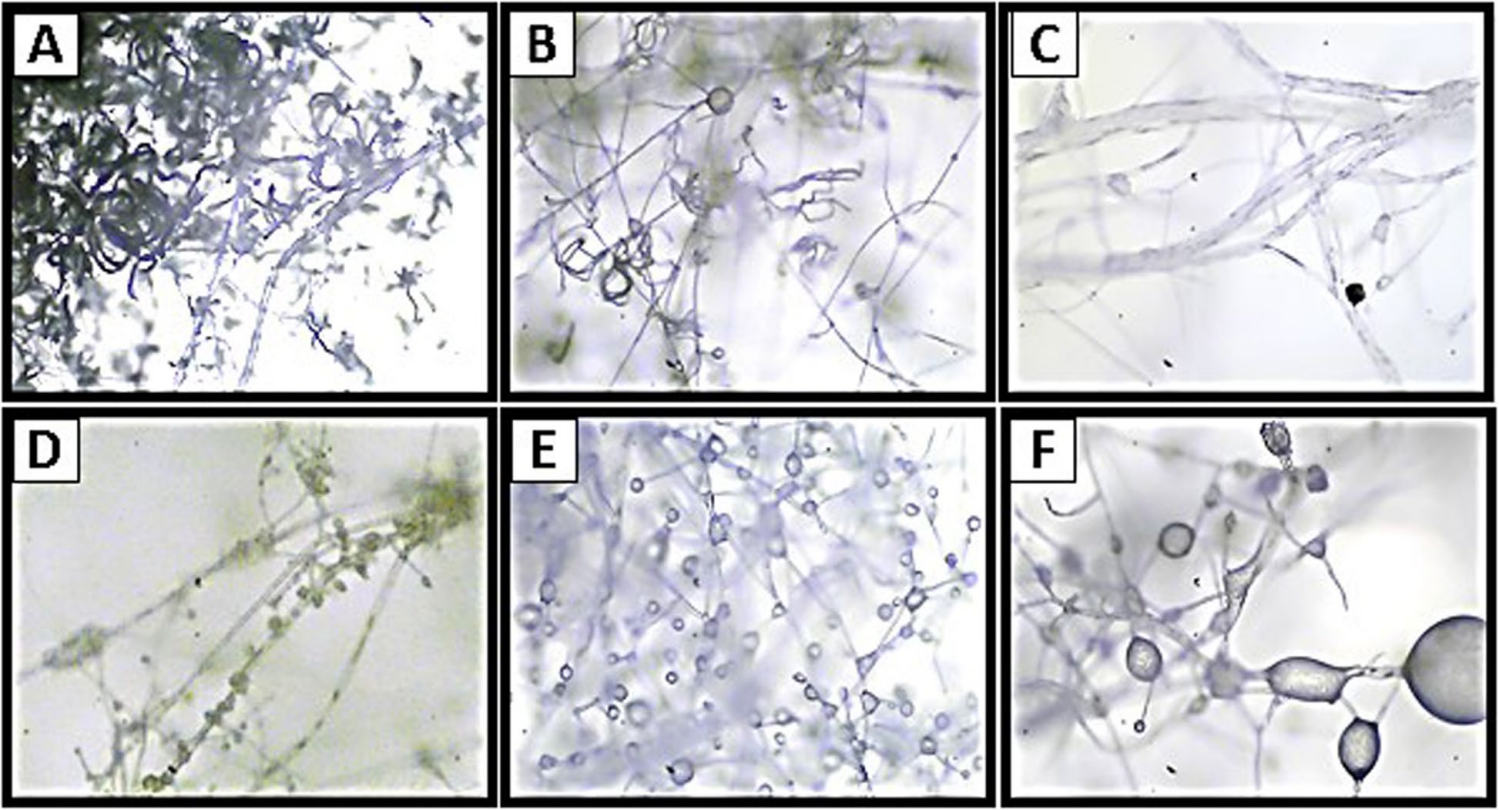
Interaction between endophytic fungi and pathogens microscopically: (**A**): endophytic fungi hyphae wrapped around pathogenic fungi hyphae, (**B**): endophytic fungal hyphae cause pathogenic hyphae to curl; (**C**): pathogenic hyphae break and disintegrate; (**D**-**F**): spores become lysed and shrive

The parasitic mechanism can be shown in the form of interaction between the endophytic microorganism and the pathogenic fungi through an abnormal cell shape change. Microscopic images show that the hyphae of endophytic fungi have the ability to wrap around the pathogenic fungi causing damage to the hyphae which is indicated by malformation of the hyphae to spiral, irregularly curved, and shortened (Fig. 4). In the case of pathogenic fungi when interacting with bacteria, the hyphae change color to transparent when observed microscopically.

## Discussion

### The diversity of endophytic and pathogenic microbes of *Boehmeria nivea*

Research on the isolation and characterization of bacterial endophytes from three tissues of ramie (*B. nivea*) was reported by Sun, et.al (2021) with the dominant species consisting of *Janibacter melonis*, *Moraxella*, and *Bacillus pumilus* in the root, stem, and leaf, respectively [33]. We found in this research that the dominant endophytic bacteria (EB) present in the ramie from Indonesia are from the genera *Curtobacterium*, *Bacillus*, *Pseudomonas*, and *Stenotrophomonas.*

In this study, information on the endophytic diversity of ramie was extended to the study of endophytic fungal (EF) groups of four plant tissues, including roots, stems, leaves, and flowers. We succeeded in isolating 11 species of rami. Young leaves showed the highest amount of EF, but in rhizome and stem shoots only the lowest amount of EF was obtained. Most isolates obtained from the leaves such as *Colletotrichum spp*., *Penicillium sp*., and *Peniophora sp*. were also shown by the isolation of EF plant in previous studies [10, 34–36]. This study is the first to report on the fungal microbiome and its potential for screening for antifungal pathogens.

The most dominant genus of EF from ramie are *Colletotrichum* and *Fusarium*, these two genera have capability to be distributed in some organs of plant. This result is in agreement with Arnold and Lutzoni who noted that the endophytic fungi that are often isolated from tropical plants are from genera *Colletotrichum* and *Fusarium* [37]*.*

In this study three species of *Colletotrichum* spp. have been isolated that can naturally play a role as pathogens, endophytes, and saprophytes [38]. Previously, *Colletotrichum* has been found in the isolation of endophytic fungi on *Citrus reticulata L* and *Paullinia cupana var. Sorbilis* [39, 40]. *C. siamense* was reported to protect nurseries, reduce the effects of mycovirus infection, and reduce lesions in *Paullinia cupana var. Sorbilis* [39].

*Fusarium* spp. has been isolated from the stem of ramie and has also been reported as a pathogen in various other plants. On the other side, *F. falciforme* has been reported as an endophyte in cassava [41]. However, in this study, *F. falciforme* strain DTO-422-H8 which was only found as an endophyte of stem root and the flower was able to act as a biocontrol by inhibiting the growth of other *Fusarium* pathogens.

Fungi pathogen from ramie is the first to be studied for its control prospect. Isolation of pathogenic fungi (FP) showed that *Fusarium* spp. and *Clonostachys rosea* are dominant on ramie stems. Fusarium are widely known as a cause of plant diseases, as observed such as banana wilt disease caused by *F. oxysporum* f.sp. cubense [42, 43], tomato wilt [44], onion [45], cucumber [46], oil palm [47], water hyacinth [48]. *F. solani* is a pathogen with symptoms of plant: stems appearing wilted and brown rot [49]; necrosis in banana seedling weeds [50]; dragon fruit plant stems (*Hylocereus* sp.); potatoes [51], chilies root rot in peanuts [52], soybeans & peas [53]. *Clonostachys rosea* has been reported commonly as a mycoparasite or saprotrophic species from soil and various plant materials [54]. However, there are a few reports of *C. rose* causing root rots in soybean in Minnesota [55] and in faba bean in Iran [19].

*Purpureocillium lilacinum* is an endophytic fungus that has also been found in *Kandelia candelia* which can protect the growth of host plants in environments with high Cu content by reducing Cu uptake [56]. Several endophytic fungi that have the ability to protect plants from pathogens have been reported such as: *Rhexocercosporium* sp from the host plant Chinese medicinal herb (*Sophora tonkinensis*); *Aspergilus* spp., *Penicillium* spp., *Fusarium* spp., and *Phoma* spp. Hist plant Eluesine coracana (Finger millet); *Phialocepala sphaeroides*, host plant (Picea abies, Nowway spruce); *Paraconiothyrium SSM001*, host plant *Taxus* spp., Yew tress; *Tricoderma hamatum* UoM, host plant *Pennisetum glaucum*, pearl millet; *Colletotricium tropicale*, host plant *Theobroma cacao*, Cacao tree; *Beauveria bassiana*, host plant *Solanum lycopersicum* and *Gossypium* spp., (tomato and cotton); *Neotyphodium coenophialum*, palnt host *Festuca arundinacea*, tall fescue; *Epichloe occultans*, plant host *Lolium multiflorum*, annual ryegrass; *Exophiala pisciphila*, *Zea mays*, maize or corn [57].

### The antagonistic activity of endophytic microbes of *Boehmeria nivea*

A total of 11 fungal isolates were tested for their antagonistic activity based on their inhibitory strength as measured by PGI value that indicated their potential to produce metabolites [30, 31]. Endophytic fungi isolated from the rhizome shoots, stem shoots, young leaf shoots, and flowers of ramie plants had antagonistic activity with PGI values in the range of 3 to 4 (strong to very strong category), especially against *Fusarium spp*., including *F. solani* isolate 324,891, *F. solani isolate* ColPat 359, and *F. oxysporum*. Fungi endophyte isolates were known to show antifungal abilities similar to *Colletotrichum gloeosporioides* which were able to produce taxol (163.4 g /l) [58] and has the potential to be a biological control agent against leaves of *Malva pusilla* and leaves of *Abutilon theophrasti* [59]. The antagonistic activity was also found in *Cladosporium tenuissimum*, which has been reported to contain anti-cancer and antimicrobial activity [60].

*Penicillium citrinum* isolate MEBP0017 has superior potency in the inhibitory spectrum on four fungi pathogens of ramie plants with a very strong ability category. *P. citrinum* is reported to produce mycotoxin nephrotoxins and several other compounds, such as tanzowaic A acid, quinolactacin, quinocitrinine, asteric acid, and compactin [61].

Therefore, the isolation of endophytic bacteria in ramie plants produced 9 bacteria isolates. The isolates were tested for antagonistic activity against 4 pathogenic fungi of ramie plants. Only five endophytic bacteria showed inhibition activity with a PGI value of 1 to 3 (weak to strong category). The antagonistic activity of endophytic bacteria against *Fusarium spp* was higher than against *Clonostachys rosea*.

Based on the results of the antagonistic test between endophytic fungi and bacteria against the pathogen *C. rosea*, it is known to have excellent mycoparasite abilities. It can act as a strong biological control against many plant pathogenic fungi, nematodes, and insects [62]. Based on the results obtained, endophytic bacterial isolates that showed the highest percentage on a scale of 3 were *Bacillus haynesii*. The inhibition of endophytic bacteria against pathogenic fungi in vitro is one indicator of its ability to suppress the growth of ramie plants pathogens. *Bacillus haynesii* strain 1605 isolated from flowers. *Bacillus haynesii* is a group of non-toxic, environmentally friendly bacteria and can be obtained in abundance for biogenic synthesis and non-pathogenic. Therefore, a study on the potential of these bacteria needs to be developed [4]. The results showed that the interaction between endophytic microbes and pathogenic fungi is through pathogenic hyphae that become abnormal or malformations (Fig. 4). It is because endophytic microbes produce antibiotic compounds that can damage and inhibit the growth of pathogens [63]. Endophytic microbes formed hooks around pathogenic hyphae before penetration or sometimes entered directly into pathogens cells. The mechanism of action of antimicrobial compounds against pathogens is by damaging cell walls, interfering with microbial cell metabolism, inhibiting cell synthesis, interfering with cell membrane permeability, inhibiting protein and cell nucleic acid synthesis in microbes [64].

In addition, we did not assess the extent of the mechanism in detail in order to identify the mode of interaction between endophytic antagonism and pathogens in plant tissues. Future research will be interesting to develop selected superior isolates in this case *Peniciilium citreneum* against plant pathogens as a fungicide application. In addition, it is possible that by connecting the metabolomic studies of ramie, other endophytic bioprospecting correlations can be seen which are very useful for reaching their potential applications in agriculture, industry, and health. However, it is also important that future studies are designed to better consider the complexity of microbial species growth and exchange under in vitro and in vivo conditions.

## Conclusions

We have identified four pathogenic fungi *Fusarium* spp., and *Clononostachys rosea* that latently interfere with the growth of ramie stems. We also isolated bacteria and endophytic fungi from the roots, stems, leaves, and flowers of ramie from Sumedang, West Java, Indonesia. A dual test in vitro can rank endophytic microbes based on their effectiveness against pathogenic fungi. In vitro antagonist screening showed that most of the isolates of fungi and endophytic bacteria were potentially effective in inhibiting the growth of pathogens. However, the isolate of *Penicillium citrinum* MEBP0017 showed a strong antagonist potential (PGI level 4) with a very active category for all the pathogenic. Our study provides more complete information on the presence of ramie endophytic bacteria and fungi and their prospects as potential biocontrol agents for the plant itself. The role of endophytic fungi is highly recommended for further development as antifungal bioproducts. In order to better understand the conditions in the field, it is also important that future research is designed for testing in a variety of environmental and in vivo conditions. Fungal endophytic microorganisms can be further explored for their use in plant breeding applications, secondary metabolites for industry, and health.

## Materials and methods

### Materials

Ramie (*Boehmeria nivea*) samples were collected from March 2021 to May 2021 at the Research Site in Jatinangor, Sumedang, West Java, Indonesia. The climate and geographical of cultivation site during the study was shown it Table 5. Data were based on Meteorology, Climatology, and Geophysical Agency (BMKG) in Sumedang [65]. Ramie samples were taken from healthy plant parts and did not show any disease symptoms to isolate the endophyte. Therefore, ramie stems showing disease symptoms were selected to obtain isolates of pathogenic microbes. Agar media in the form of PDA (Potato Dextrose Agar) and NA (Nutrient Agar) OXOID were prepared in a petri dish free of contamination.

**Table 5:** Climate and geographical condition of Sumedang, West Java, Indonesia

Parameter	Value
Elevation of the location (masl)	721
Average temperature (^o^C)	27.92
Average humidity (%)	87.04
Total rainfall (mm)	380

### A sampling of endophyte and pathogenic isolates

The initial determination of plant species was carried out at the Taxonomy Laboratory of the Department of Biology, FMIPA, Padjadjaran University. Ramie samples taken from healthy plant parts and showing no disease symptoms were used to obtain isolates of endophytic microorganisms, including endophytic fungi (EF) and endophytic bacteria (EB). Ramie stems showing symptoms of the disease were selected as research samples to obtain pathogenic microbial isolates. A total of 10 – 15 parts of young plant organs, including rhizome shoots (RS), stem shoots (SS), young leaf shoots (YLS), and flowers (FS), were taken randomly from the ramie plant and put into clean polyethylene bags, which were immediately brought to the laboratory.

### Sample sterilization

A surface sterilization procedure cleaned each part of the sample plant organs to remove dirt and epiphytic microbes. Each plant tissue was cut with a sterile knife into 1 cm segments and was washed under running water. After that, the surface was sterilized by immersing the samples in 75% ethanol for 2 min, 5.3% sodium hypochlorite for 5 min, 75% ethanol for 30 s, and finally, rinsed with distilled water for 1 min. After that, the samples were dried on sterile filter paper [66].

This procedure refers to Potshangbam et al. (2017) method with slight modifications [67]. Isolation of endophytic microbes was carried out using the direct planting method. Each organ of the plant sample cut with a size of 1 × 1 cm in a petri dish containing PDA or NA isolation media that was given antibiotics (50 mg/l). Control media was not overgrown with contaminant fungi or bacteria during incubation [68]. Each plate was incubated for ± 14 days at a temperature of 27–29 °C.

Only endophytic microbes that grew in the sample were isolated onto a new growing medium plate by streak technique. The cultures were incubated at 27 °C for 3–5 days to separate single colonies, which were then transferred to a slanted agar medium in test tubes to serve as stock cultures. These cultures were incubated and stored as pure cultures available for the next stage.

### Pathogenic fungi isolation

Pathogenic fungi were isolated from diseased parts of ramie stems. Surface sterilization of diseased samples was washed under running water for 10 min. Surface sterilization is carried out in a sterile room. After that, the sample was dried on sterile filter paper Samples of stems that have been cut into pieces with a size of ± 4 × 1 cm are placed in a petri dish containing PDA isolation media to which chloramphenicol (50 mg/1) hour has been added. The fungi culture was placed at 25 for 2 × 24 h [66, 69, 70].

### Morphotype identification

Macroscopic observations parameters of microbial morphology were based on Gandjar et al. including the front and backside colony surfaces color, the presence of a radial line from the center of the colony to the edge of the colony, the presence of concentric shapes forming a circle on the inside of the colony, the surface texture, and topography of the colony [71]. In microscopic observation of microbial morphology, fungal culture was carried out using the moist chamber method. The parameters were hyphae of the septum, growth, color, and conidia ranging from shape and color. The moist chamber results were observed using microscope type of Binocular RCC Multimedia (Digimi 107MT).

### DNA isolation

DNA extraction was carried out using the iNtRON i-genomic BYF DNA Extraction Mini Kit. The isolates were re-grown on liquid media (PDB). The grown isolate was taken on the surface of the liquid medium (1 × 1 29 cm) and put into a 2 mL tube containing 200 µL of distilled water. The isolate was mashed using Qiagen Tissue Ruptor. Then, 100 L Buffer MP was added, and samples were vortexed for 30 s. Samples were incubated at 37 °C for 15 min and centrifuged at 13,000 rpm for 1 min. After that, the supernatant of the samples was removed. 200 µL Buffer MG, 20 µL Proteinase K, and 5 µL RNase were added to the sample and vortexed. The sample was incubated at 65 °C for 30 min and 250 µL of MB buffer was added to the sample. 250 µL of 80% ethanol was added to the sample. Next, the sample was transferred to a spin column and put into a 2 mL collection tube. Samples were centrifuged at 13,000 rpm for 1 min. The spin column was transferred to a new 2 mL collection tube, then 700 µL of Buffer MW was added to the spin column and centrifuged at 13,000 rpm for 1 min. The supernatant contained in the collection tube was removed and the spin column was reinserted into the collection tube. Centrifugation was carried out again by adding 50 L Buffer ME under the same conditions. Furthermore, the samples obtained were used for the next step, including amplification, purification, and DNA sequencing.

### DNA sequence analysis and phylogenetic

The resulting DNA sequences were aligned using the MUSCLE software embedded in MEGA X [72] to trim and edit to obtain the complete sequence. Homology searches were performed using the BLASTn program in the NCBI GenBank database (https://blast.ncbi.nlm.nih.gov/Blast.cgi). A suitable DNA substitution model for the ITS Genetic Analyzer ABI 3130 XL gene was assessed using the “find best DNA/Protein Models (ML)” function in the MEGA X software to obtain the nucleotide sequence of the ITS and 18S rDNA regions. It implements the maximum likelihood statistical method (ML) to test its goodness against several evolutionary models. According to the estimated values of all parameters for each model, the model that best fits the dataset of the ITS sequence is the generalized time-reversible (GTR) and gamma-distributed (+ G) model with site invariance (+ I) (= GTR + G + I) model. The ML tree was constructed using MEGA X with all positions containing gaps and missing data entered for analysis. Clade support is calculated based on 1,000 bootstrap re-sampling.

### Endophytic microbial antagonist test against ramie pathogenic fungi

Observations on the antagonistic mechanism of ramie endophytic bacteria and fungi against ramie pathogenic fungi were carried out by analyzing four types of antagonistic mechanisms. According to Skidmore and Dickinson, observation of the antagonist mechanism was carried out through direct observation in dual cultures and by taking 1 cm × 1 cm hyphae pieces in the contact area of antagonistic microbes and pathogens, then it placed on a slide to determine observed under a microscope.

The antagonistic mechanisms include type of competition, If the antagonist fungi colony covered the pathogenic colony and the growth of the antagonist fungus was faster to fill the petri dish. At the contact area, the pathogenic hyphae undergo lysis; antibiosis, when an empty zone is formed between the pathogenic fungi and the antagonist fungi, there is a change in the shape of the pathogenic hyphae, and the pigment is produced on the lower surface of the antagonist fungi colonies; parasitism, if the antagonist fungi grow on the hyphae of the pathogen, in the contact area the hyphae of the antagonist fungi are found wrapped around the hyphae of the pathogen and lysis occurred [32].

The percentage growth of inhibitory (PGI) of endophytic fungi as antagonists was calculated using the formula:

$$\mathrm{PGI}\;(\%)=PGI\;\left(\%\right)=\frac{Rk-R1}{Rk}x100\%$$

PGI = *Percentage Growth Inhibition* (%).

Rk = Growth distance of ramie pathogenic fungi control from the point of inoculation to the edge of the colony.

R1 = Radius of pathogenic fungi colonies whose growth direction is close to antagonistic endophytic isolate colonies.

According to Živković et al., PGI is categorized as growth inhibition on a scale of 0 to 4. The percentage of inhibition categories: scale 0 for no inhibition is categorized very weak; scale 1 for 1%—25% inhibition is weak; scale 2 for 26% -50% inhibition is intermediate; scale 3 for 51%—75% inhibition is strong; scale 4 for 76% -100% inhibition is very strong [73].

## Data Availability

All data generated or analysed during this study are included in this published article.

## Abbreviations

EF: Endophytic fungi
EB: Endophytic bacteria
PF: Pathogenic fungi
RS: Rhizome shoots
SS: Stem shoots
YLS: Young leaf shoots
FS: Flowers
PGI: Percentage growt inhibition
